# Selective expression of KCNA5 and KCNB1 genes in gastric and colorectal carcinoma

**DOI:** 10.1186/s12885-020-07647-x

**Published:** 2020-12-02

**Authors:** Azer Farah, Maria Kabbage, Salsabil Atafi, Amira Jaballah Gabteni, Mouadh Barbirou, Mouna Madhioub, Lamine Hamzaoui, Mousadak Azzouz Mohamed, Hassen Touinsi, Asma Ouakaa Kchaou, Emna Chelbi, Samir Boubaker, Rahma Ben Abderrazek, Balkiss Bouhaouala-Zahar

**Affiliations:** 1 Laboratory of Venoms and Therapeutic Biomolecules, LR16IPT08 Institute Pasteur Tunis, Tunis Belvédère- University of Tunis El Manar, 13 Place Pasteur, BP74, Tunis, Tunisia; 2 Biomedical Genomics and Oncogenetics Laboratory, LR11IPT05 Institut Pasteur de Tunis, Université Tunis El Manar, Tunis, Tunisia; 3 Laboratory of Human and Experimental Pathology, Institute Pasteur Tunis, University of Tunis El Manar, Tunis, Tunisia; 4 Center for Biomedical Informatics, Department of Health Management and Informatics, School of Medicine, University of Missouri, Columbia, MO USA; 5 Gastroenterology Department, Mohamed Tahar Maamouri Hospital, 8000 Nabeul, Tunisia; 6 Surgical Department, Mohamed Tahar Maamouri Hospital, 8000 Nabeul, Tunisia; 7 Gastroenterology Department, Habib Thameur Hospital, Tunis, Tunisia; 8 Pathology Department, Mohamed Tahar Maamouri Hospital, 8000 Nabeul, Tunisia; 9 Medical School of Tunis, University of Tunis El Manar, Tunis, Tunisia

**Keywords:** Gastric cancer, Colorectal cancer, Kv1.5, Kv2.1, Gene expression, Intracellular localisation, Cancer diagnosis

## Abstract

**Background:**

Gastric and colorectal cancers are the most common malignant tumours, leading to a significant number of cancer-related deaths worldwide. Recently, increasing evidence has demonstrated that cancer cells exhibit a differential expression of potassium channels and this can contribute to cancer progression. However, their expression and localisation at the somatic level remains uncertain. In this study, we have investigated the expression levels of KCNB1 and KCNA5 genes encoding ubiquitous Kv2.1 and Kv1.5 potassium channels in gastric and colorectal tumours.

**Methods:**

Gastric and colorectal tumoral and peritumoral tissues were collected to evaluate the expression of KCNB1 and KCNA5 mRNA by quantitative PCR. Moreover, the immunohistochemical staining profile of Kv2.1 and Kv1.5 was assessed on 40 Formalin-Fixed and Paraffin-Embedded (FFPE) gastric carcinoma tissues. Differences in gene expression between tumoral and peritumoral tissues were compared statistically with the Mann-Whitney U test. The association between the clinicopathological features of the GC patients and the expression of both Kv proteins was investigated with χ2 and Fisher’s exact tests.

**Results:**

The mRNA fold expression of KCNB1 and KCNA5 genes showed a lower mean in the tumoral tissues (0.06 ± 0.17, 0.006 ± 0.009) compared to peritumoral tissues (0.08 ± 0.16, 0.16 ± 0.48, respectively) without reaching the significance rate (*p* = 0.861, *p* = 0.152, respectively). Interestingly, Kv2.1 and Kv1.5 immunostaining was detectable and characterised by a large distribution in peritumoral and tumoral epithelial cells. More interestingly, inflammatory cells were also stained. Surprisingly, Kv2.1 and Kv1.5 staining was undoubtedly and predominantly detected in the cytoplasm compartment of tumour cells. Indeed, the expression of Kv2.1 in tumour cells revealed a significant association with the early gastric cancer clinical stage (*p* = 0.026).

**Conclusion:**

The data highlight, for the first time, the potential role of Kv1.5 and Kv2.1 in gastrointestinal-related cancers and suggests they may be promising prognostic markers for these tumours.

## Background

Gastrointestinal-related cancers, especially gastric cancer (GC) and colorectal cancer (CRC), form the vast majority of overall malignant occurrences [1], with GC and CRC cases forming a major contribution to the global cancer burden [2, 3]. According to The Global Burden of Disease Study in 2017, CRC and GC were ranked within the top 5 global cancers in terms of cancer prevalence [4]. Recently, CRC and GC morbidity and mortality have increased in Tunisia. In 2018, an estimated 401 new GC cases were reported [5], whereas CRC cases reached a rate of 6.3/100,000, leading these cancers to become diseases of high public health concern [5, 6].

CRC and GC are usually diagnosed at an ill-timed and advanced stage due to the absence of national early screening programs and a lack of specific symptoms or reliable diagnostic and prognostic biomarkers [7]. However, GC tumorigenesis differs from CRC by different factors and molecular pathways, and involves a heterogeneous complexity [8]. Notwithstanding, it remains challenging to identify the mechanism of association between these two cancers. Therefore, identifying new biomarkers useful for early prognosis and diagnosis, and additional therapeutic targets for GC and CRC, is of great importance [9].

Ion channels (i.e. Na+, K+) are pore-forming proteins, composed by two to four units of six transmembrane segments, that allow ions across membranes and have been proven to be novel targets for cancer therapy [10]. In particular, voltage-dependent K+ (Kv) channels have recently received increased attention [11]. Although it is widely believed that Kv channels control proliferation ubiquitously by allowing cell cycle progression, their expression is altered in many cancers, and their participation, as well as their use as tumour markers, deserves further attention [12].

Currently, Kv channels have been proposed as candidates for modulating the effect of therapeutic drugs, due to their capacity for reducing tumour cell progression [13, 14] and their differential expression in human cancers [15]. Several studies have been performed on prototypes of cell lines and only a few studies have compared the properties of Kv channels in normal epithelial cells versus tumour cells [12]. Furthermore, research shows that the most important isoforms for the proliferation, activation, migration and apoptosis of tumour cells are Kv1.3, Kv1.5, Kv2.1, Kv41, Kv9.3, Kv10.1 and Kv11.1 [16]. However, the mechanisms by which these six-transmembrane Kv channels contribute to malignant transformation and progression are still not fully understood [17]. Moreover, the correlation between Kv gene expression and tumour staging has not yet been clearly demonstrated [11, 18]. Recently, KCNB1 polymorphisms were correlated to CRC treatment and patient’s outcome in Tunisia population [19]. Kv channels are mostly expressed in the plasma membrane, and diverse Kv channels also exist in intracellular organelles [20–24]. Therefore, one of the major challenges is to determine whether the intracellular localisation of Kv channels might be involved in malignant transformation [12, 25, 26]. Among voltage dependent potassium channels, the Kv1.5 and Kv2.1 subtypes are known to be broadly expressed in a variety of tissues and their altered expression has been detected in several cancers, especially in GC and CRC cell lines [12, 27]. The subtype Kv2.1, encoded by the KCNB1 gene, has been well characterised at the functional level and its role in several cancers, including gastric and uterine cancers, has been demonstrated [27]. Kv2.1 occasionally forms complexes with other voltage-gated potassium alpha-subunits (i.e. Kv9.3), with its silencing potently inhibiting proliferation in human colon cells, suggesting an important role in gastro-intestinal related cancers [28]. Likewise, the Kv1.5 channel, encoded by the KCNA5 gene, is reported to have a low/medium expression on the surface of colorectal and stomach cancer cell lines (provided by The Human Protein Atlas).

However, the level of KCNA5 and KCNB1 gene products in human GC and CRC tissues has been poorly investigated to date. In the present study, we examined the quantitative mRNA expression profiles of KCNA5 and KCNB1 in tumour and peritumoral tissues and investigated their protein expression levels by immunostaining. We showed non-membranous immunoreactivity, suggesting a moderate cytoplasmic localisation in gastric carcinoma.

## Methods

### Study subjects

This retrospective study was conducted between January 2006 and May 2017, at the Pathology Department of the University Hospital, Nabeul and Gastroenterology Department of Habib Thameur Hospital, Tunis in Tunisia. A total of 75 subjects with CRC or GC were recruited: 59 patients had GC and 16 patients had CRC. Tissue samples were obtained from endoscopy (colonoscopy or fibroscopy) or during surgery (colectomy or gastrectomy). Clinico-pathological parameters, such as staging, tumour site, histological type and tumour differentiation, were evaluated by reviewing medical charts and pathological reports. The clinical stage of the tumour was assigned according to the TNM classification of the UICC: the tumour infiltration degree (T), Node metastases (N) and Metastases (M). The study cohort was pooled into two subgroups according to tumour staging: with stage I-II as an early/localised stage and stage III-IV as advanced/metastatic stage. The missing data for tumour clinical stage corresponds to non-resectable patients with loco-regional metastases and in whom the TNM stage was hard to identify from biopsies. None of the patients had undergone any pre-operative treatment.

### cDNA preparation

Somatic DNA, RNA and protein preparation was performed using the All Prep DNA/RNA/Protein Mini Kit QIAGEN according to the manufacturer’s instructions (Qiagen GmbH, Hilden, Germany). First-strand cDNA was synthesised from total RNA with oligo-dT primers using Invitrogen™ SuperScript™ II Reverse Transcriptase (Thermo Fisher Scientific, Newington, USA).

### SYBR green Q-PCR

The mRNA expression levels of KCNB1 and KCNA5 in tumour and peritumoral tissues was evaluated by quantitative PCR, using a Light Cycler 480 (Roche, Mannheim, Germany) with SYBR Green mix (Roche, Mannheim, Germany). The forward and reverse primers for KCNB1 and KCNA5 genes were, respectively: 5′- CTGTCTGAAACCAGCTCAAG-3′, 5’GTCTTCCAACTGCTGAACG-3′ 5′-TGAGTTCAGGGATGAACGTG-3′ and 5′-GGTCTCCACGATGAAGAAGG-3′. The PCR product was visualised with 2% agarose gel electrophoresis. All samples were tested in duplicate and run on the same plate. To control for the expression level of KCNB1 and KCNA5 genes in the reaction mix, data were normalised to the expression of the endogenous gene Glyceraldehyde-3-Phosphate Dehydrogenase GAPDH (Forward and reverse primer: 5′-TGCACCACCAACTGCTTAGC-3′ and 5’GGCATGGACTGTGGTCATGAG-3′). To calculate relative quantification values, a threshold cycle (Ct) at which a statistically significant increase in fluorescence occurs was derived from the resulting qPCR profiles of each sample. Ct is a measure of the amount of template present in the starting reaction. Ct values for GAPDH were subtracted from those of the corresponding KCNB1 and KCNA5 levels, resulting in ∆Ct. This is to essentially normalise the gene of interest to a gene which is not affected by the experimental conditions. The relative quantification value of KCNB1 and KCNA5 mRNA levels was expressed as 2^-∆Ct^, giving the fold change in gene expression.

### Immunohistochemistry and IRS scoring

Prepared tissue sections were examined by two independent pathologists and only adenocarcinoma cases were selected for the immunostaining assay. Staining was carried out on formalin-fixed, paraffin-embedded samples using the anti-Kv1.5 polyclonal antibody [29] and Kv2.1 monoclonal antibody [30] (Abcam, Cambridge, UK) at a final dilution of 1:300, according to a well-established protocol [31]. The intensities of the immunohistochemical reactions were visually estimated independently by two pathologists. In order to evaluate the expression profile of the analysed proteins in tissue sections, a semi-quantitative scale of the Immuno-Reactive Score (IRS) was adopted. The (IRS) gives a range of 0–12, as a product of multiplication between the proportion of positive cells score (0–4) and the staining intensity score (0–3) [32, 33].

### Statistical analysis

Statistical analysis was performed using GraphPad Prism software 5.0 (GraphPad Software, Inc., San Diego, CA). The average of KCNB1 and KCNA5 gene expression levels were compared, between tumoral and peritumoral tissues of patients with GC and CRC, using the non-parametric Mann-Whitney U test. Correlations between the expression of KCNB1 and KCNA5 genes were analysed using Spearman’s rank correlation. The association between clinicopathological features of the GC patients and Kv2.1 and Kv1.5 protein expression was analysed by the χ2 test (or Fisher’s exact test when *n* < 5). *P* values < 0.05 were considered statistically significant.

## Results

This study was conducted between June 2015 and September 2016. All the recruited patients give their written consent to take part and the study procedure acquired prior approval from the local ethics committee (ref. ISA/2016/02). Clinico-pathological data were analysed anonymously.

### Clinico-pathological features of patients

Among the 75 recruited patients who underwent clinical examination and had a confirmed diagnosis, 59 subjects had GC and 16 had CRC. The characteristics of CRC and GC patients (16 and 19 subjects, respectively) investigated for KCNA5 and KCNB1 gene expression are presented in Table 1. Most GC and CRC cases presented with adenocarcinoma (100%), were poorly differentiated (GC (59.90%) vs CRC (50%)) and were at an advanced/metastatic stage (GC (52.64%) vs CRC (81.25%)). Immunohistochemistry assays were carried out on tissue samples from 40 patients with GC. The clinico-pathological features of GC patients are shown in Table 2. Most GC cases presented as diffuse adenocarcinoma type (50%), were poorly differentiated (57.5%) and at early/localised stage (60%).

**Table 1:** Clinical and pathological characteristics of patients with GC and CRC

Parameters	GC Cases ***N*** = 19 (%)	CRC Cases ***N*** = 16 (%)
**Age (***years***)** ^**a**^	49.2 ± 16.04	59,75 ± 11,96
**Gender (***Males / Females***)**	11/8 (57.90/ 42.10)	8/8 (50/ 50)
**Differentiation**		
*Poor*	11 (59.90)	8 (50)
*Moderate*	4 (21.05)	6 (37.50)
*Well*	4 (21.05)	2 (12.50)
**Histological metastasis**		
*Positive*	4 (21.05)	3 (18.75)
*Negative*	15 (78.95)	13 (81.25)
**TNM classification**		
*Early/localized stage (I, II)*	9 (47.36)	3 (18.75)
*Advanced/metastatic stage (III, VI)*	10 (52.64)	13 (81.25)

*TNM* Tumor, Nodes, Metastases according to the American Joint Committee on Cancer (AJCC), ^a^ mean ± standard deviation

**Table 2:** Expression of Kv2.1 and Kv1.5 proteins and clinico-pathological features of patients with GC

Parameters	Kv2.1 protein, *N* = 40 (%)				P ^a^	Kv1.5 protein, *N* = 40 (%)				P ^a^
	Negative	Mildly Positive	Moderately Positive	Strongly Positive		Negative	Mildly positive	Moderately positive	Strongly positive	
Gender										
Male	8 (20)	3 (7.5)	10 (25)	5 (12.5)	0.36	6 (15)	5 (12.5)	11 (27.5)	4 (10)	0.64
Female	7 (17.5)	3 (7.5)	3 (7.5)	1 (2.5)		6 (15)	2 (5)	4 (10)	2 (5)	
Age (*years*)										
< 50	5 (12.5)	2 (5)	6 (15)	2 (5)	0.89	6 (15)	2 (5)	5 (12.5)	2 (5)	0.75
≥ 50	10 (25)	4 (10)	7 (17.5)	4 (10)		6 (15)	5 (15.5)	10 (25)	4 (10)	
Lauren classification										
Diffuse	8 (20)	3 (7.5)	7 (17.5)	2 (5)	0.78	7 (17.5)	4 (10)	7 (17.5)	2 (5)	0.94
Intestinal	5 (12.5)	3 (7.5)	3 (7.5)	3 (7.5)		4 (10)	2 (5)	5 (12.5)	3 (7.5)	
Mixed	2 (5)	0 (0)	3 (7.5)	1 (2.5)		1 (2.5)	1 (2.5)	3 (7.5)	1 (2.5)	
Tumor localization										
Antro-Pyloric	4 (10.5)	0 (0)	4 (10.5)	4 (10.5)	0.09	1 (2.6)	4 (10.5)	5 (13.2)	2 (5.3)	0.36
Cardia	1 (2.6)	2 (5.3)	1 (2.6)	0 (0)		1 (2.6)	1 (2.6)	2 (5.3)	0 (0)	
Antro-fundic	9 (23.7)	4 (10.5)	8 (21.1)	1 (2.6)		9 (23.7)	2 (5.3)	7 (18.4)	4 (10.5)	
TNM classification										
Early/localized stage (I, II)	6 (19.4)	4 (12.9)	10 (32.3)	4 (12.9)	**0.026**	4 (12.9)	5 (16.1)	11 (35.5)	4 (12.9)	0.056
Advanced/metastatic stage (III, VI)	6 (19.4)	0 (0)	0 (0)	1 (2.6)		4 (12.9)	1 (3.2)	0 (0)	2 (6.5)	
Differentiation										
Poorly	10 (25)	3 (7.5)	8 (20)	2 (5)	0.41	8 (20)	5 (12.5)	8 (20)	2 (5)	0.48
Moderately	3 (7.5)	2 (5)	4 (10)	1 (2.5)		2 (5)	0 (0)	5 (12.5)	3 (7.5)	
Well	2 (5)	1 (2.5)	1 (2.5)	3 (7.5)		2 (5)	2 (0)	2 (5)	1 (2.5)	
Tumour infiltration										
pT1-T2	4 (12.9)	3 (9.7)	6 (19.4)	3 (9.7)	0.41	2 (6.5)	3 (9.7)	8 (25.8)	3 (9.7)	0.24
pT3-T4	8 (25.8)	1 (3.2)	4 (12.9)	2 (6.5)		6 (19.4)	3 (9.7)	3 (9.7)	3 (9.7)	
Metastasis										
Absent	11 (27.5)	6 (15)	13 (32.5)	6 (15)	0.06	9 (22.5)	7 (17.5)	15 (37.5)	5 (12.5)	0.087
Present	4 (10)	0 (0)	0 (0)	0 (0)		3 (7.5)	0 (0)	0 (0)	1 (2.5)	

*TNM* Tumor, Nodes, Metastases according to the American Joint Committee on Cancer (AJCC), ^**a**^ Pearson chi square (categorical variables)

### Relative quantification analysis

**Fig. 1:**
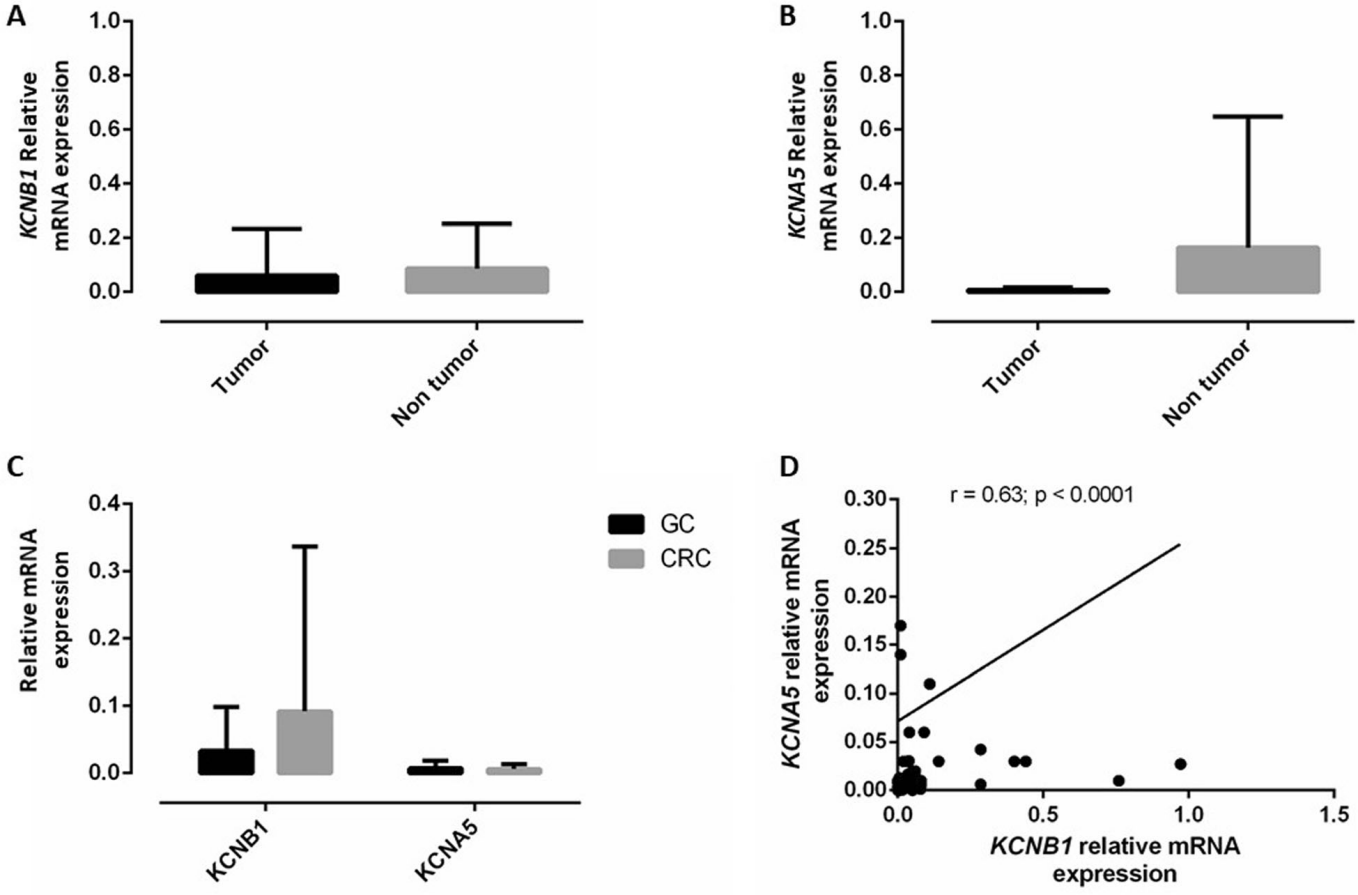
Relative mRNA expression of KCNB1 and KCNA5 in tissues from patients with GC and CRC, determined by quantitative PCR. Histograms show mRNA levels of KCNB1 (**a**) and KCNA5 (**b**) genes in 35 tumour tissues compared with their surrounding peritumoral tissues from patients with GC and CRC, expressed with the 2^-ΔCT^ method. *P*-values are based on the Mann–Whitney U-test (*p < 0.05). **c** The histogram shows the copy number variations of the KCNB1 and KCNA5 genes in tumour tissues from GC (*n* = 19) and CRC (*n* = 16) patients, expressed with the 2^-ΔCT^ method. Vertical bar graphs represent the means and the error bars represent the standard deviation calculated for each group. **d** Correlation between relative gene expression of KCNB1 and KCNA5 within CRC and GC samples (tumour and peritumoral tissue). Spearman rank correlation (r) and *p* values are indicated.

The comparison between both groups regarding KCNB1 and KCNA5 mRNA-fold expressions is shown in Fig. 1 A and B. The expression data showed that KCNB1 and KCNA5 genes have a homogenous expression level in tumour and peritumoral tissues. The mean value of KCNB1 mRNA fold expression was lower in tumour tissues (0.06 ± 0.17) compared to peritumoral tissues (0.08 ± 0.16), and the mean value of KCNA5 mRNA fold expression among tumour tissues (0.006 ± 0.009) was lower than that of the normal group (0.16 ± 0.48); however, this did not reach the level of statistical significance (*p* = 0.861 and *p* = 0.152, for KCNB1 and KCNA5, respectively).

Notably, the average of the KCNB1 gene copy numbers was 0.033 (range, 0.000 to 0.118) and 0.092 (range, 0.000 to 0.314), for GC and CRC tumour tissues, respectively. For KCNA5, the mean gene copy number was 0.007 (range, 0.000 to 0.042) and 0.005 (range, 0.000 to 0.020) for the GC and CRC tumour tissues, respectively (Fig. 1c). Regarding the correlation between KCNB1 and KCNA5 mRNA fold expressions in the studied groups, KCNB1 mRNA fold expression had a positive significant correlation with KCNA5 mRNA fold expression in tumour and peritumoral tissues (*r* = 0.63; *p* = 0.00002) (Fig. 1d).

### Immuno-detection of Kv2.1 and Kv1.5 channels

**Fig. 2:**
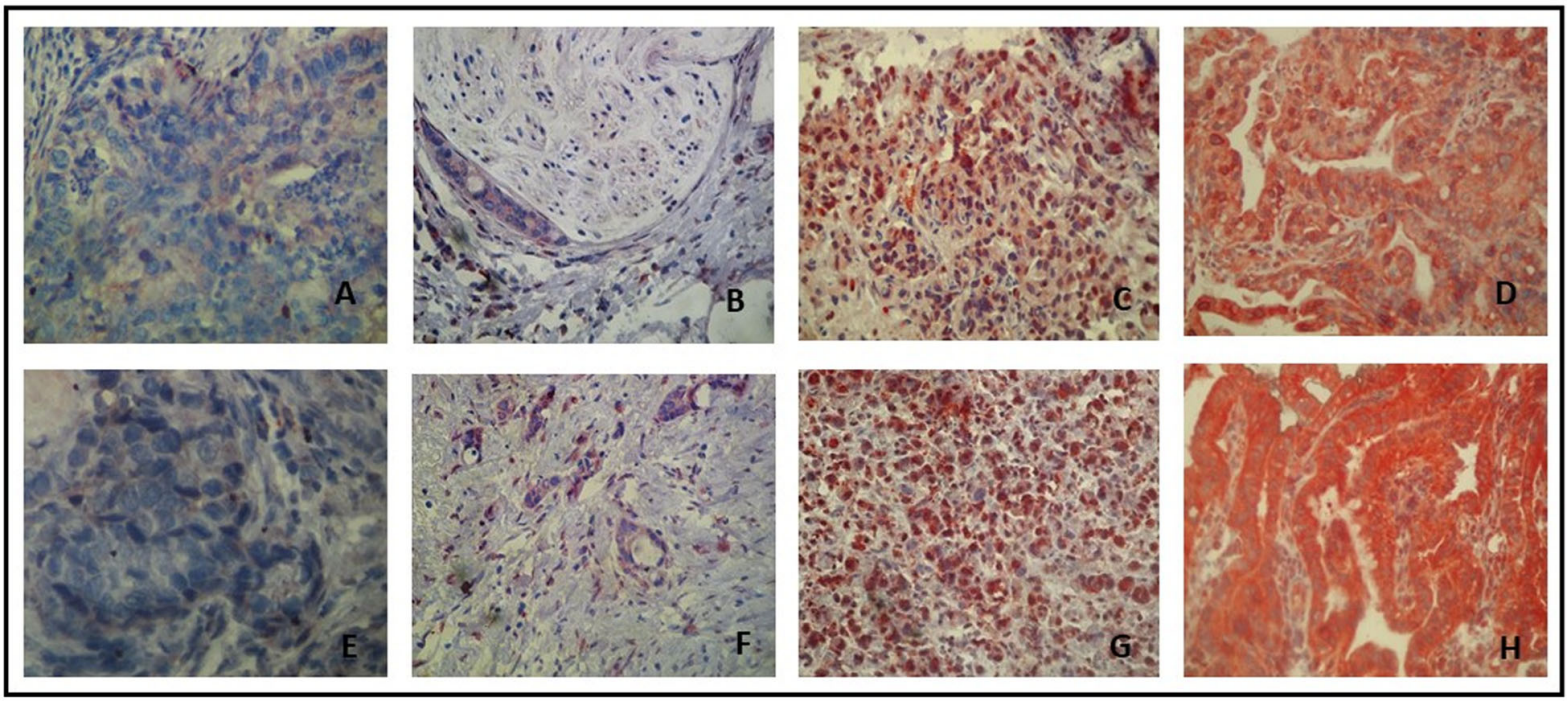
Immunostaining of Kv2.1 and Kv1.5 in gastric tumour cells. Intensity of a cytoplasmic staining of Kv2.1 (**a**, **b, c** and **d**) and Kv1.5 (**e, f, g** and **h**) in gastric tumour cells, was achieved using specific antibodies raised against the C-terminal of the Kv2.1 and Kv1.5 alpha subunits. Negative staining of Kv2.1 (**a**) and Kv1.5 (**e**) (GX400). Low positive staining of Kv2.1 (**b**) and Kv1.5 (**f**) (GX200). Moderately positive staining of Kv2.1 (**c**) and Kv1.5 (**g**) (GX200). Strongly positive staining of Kv2.1 (**d**) and Kv1.5 (**h**) (GX200)

**Fig. 3:**
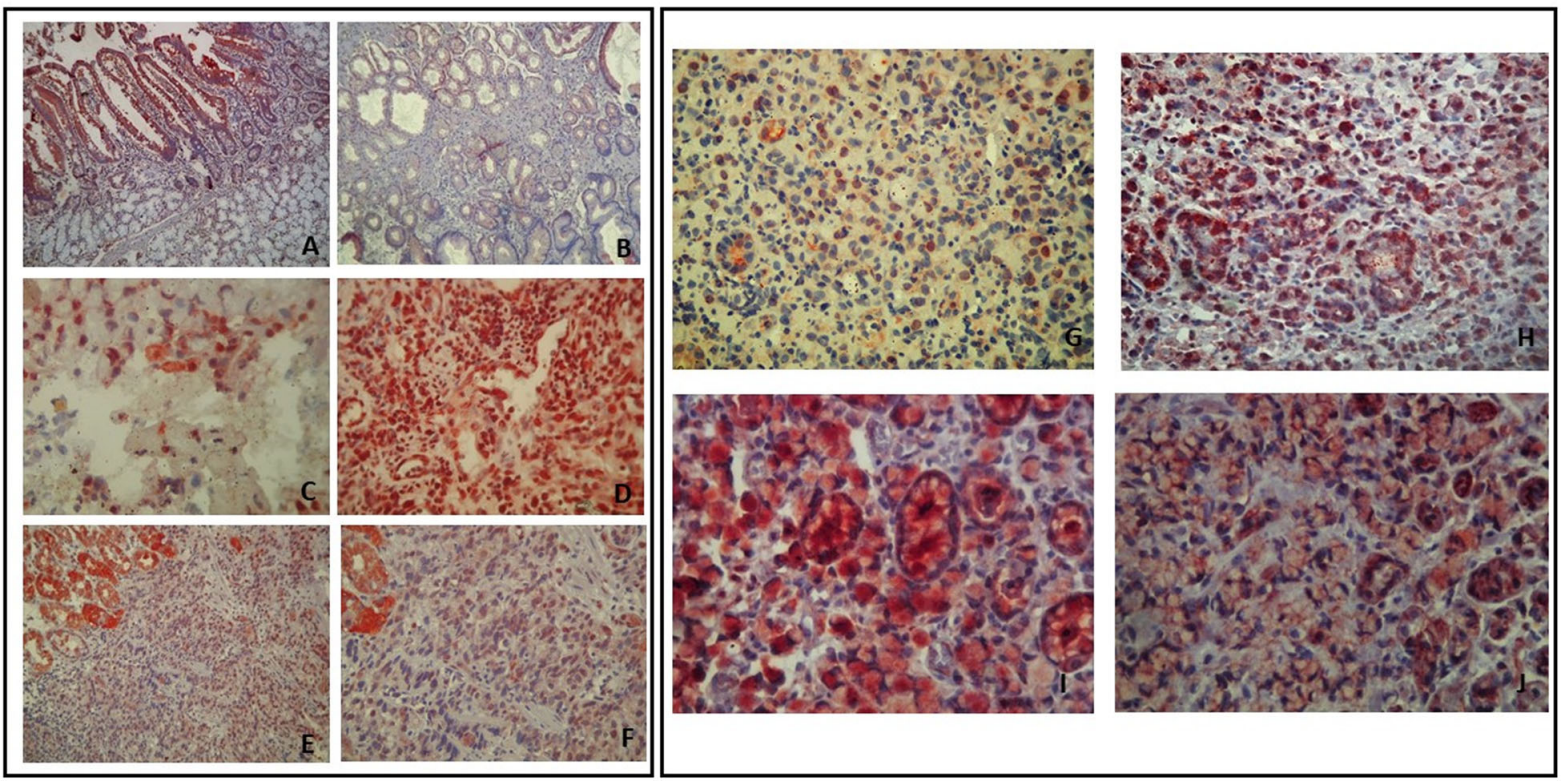
Staining of Kv2.1 and Kv1.5 in inflammatory and peritumoral gastric cells. Staining of Kv2.1 (**a** and **c**) and Kv1.5 (**b, d, e** and **f**) in inflammatory (on the right) and peritumoral gastric cells (on the left), achieved using specific antibodies raised against the C-terminal of the Kv2.1 and Kv1.5 alpha subunits. **a** Positive staining of Kv2.1 in peritumoral cells (GX100). **b** Positive staining of Kv1.5 in peritumoral cells (GX100). **c** Nuclear localisation of Kv2.1 in inflammatory cells (GX400); **d** Cytoplasmic and nuclear localisation of Kv1.5 in inflammatory cells (GX400). **e** and **f** Simultaneous staining of Kv1.5 in tumour and peritumoral cells (GX100) and (GX200). Localisation of staining for Kv2.1 (**g** and **h**) and Kv1.5 (**i** and **j**) in gastric cancer cells (on the right). **g** Cytoplasmic and nuclear co-localisation of Kv2.1 (GX200); **h** Cytoplasmic localisation of Kv2.1 (GX200); **i** Cytoplasmic localisation of Kv1.5 protein (GX400); **j** Cytoplasmic and membranous co-localisation of Kv1.5 (GX200)

The immuno-detections were achieved using specific antibodies raised against the C-terminal of the Kv2.1 and Kv1.5 alpha subunits (Abcam, Cambridge, UK). Specific Kv2.1 and Kv1.5 immunostaining was detected in epithelial and inflammatory cells (Fig. 3a, B, C and D). The Kv2.1 and Kv1.5 proteins were present in most of the GC cases (*n* = 40) 85 and 87.5% respectively, with different intensities of staining (Fig. 2). We found a dominant cytoplasmic localisation of Kv2.1 in 26 of the positive cases (65%). In addition to this cytoplasmic localisation, 4 positive cases (10%) presented with a nuclear expression of Kv2.1. The remaining positive cases (10%) showed an exclusive nuclear staining (Fig. 3g and h). For the Kv1.5 protein, the immunostaining was predominantly cytoplasmic in 29 positive cases (72.5%). However, a cytoplasmic/membranous co-localisation was noted in 6 positive cases (15%) (Fig. 3i and j). Kv1.5 and Kv2.1 immunostaining was more intense in peritumoral gastric mucosa compared to tumour cells (Fig. 3a, b, e and f).

### Correlation between Kv1.5 and Kv2.1 staining and clinico-pathological parameters

The expression of Kv2.1 in tumour cells showed a significant association with early/localised clinical stages (*p* = 0.026). However, Kv1.5 protein expression showed a borderline association with the GC early/localised stages (*p* = 0.056). No significant associations were noted between Kv2.1 and Kv1.5 expression profiles and the clinico-pathological parameters tested (Table 2).

## Discussion

In the epithelial cells of the gastrointestinal tract, Kv channels have a large molecular diversity and are involved in a variety of important biological and physiological functions. Kv channels are known to be associated with the control of cell differentiation, proliferation and apoptosis [17, 34]. In this report, we assessed the Kv expression profiles in Tunisian CRC and GC patients. To the best of our knowledge, this is the first study investigating both KCNA5 and KCNB1 genes in gastrointestinal-related tumour tissues, at both RNA and protein phases of gene expression.

The data revealed no significant differences in expression between KCNA5 and KCNB1 in the tumour tissues as compared to the peritumoral tissues. Moreover, the variation of KCNB1 and KCNA5 copy numbers showed a tendency to down-regulation in GC and CRC as compared to GAPDH gene expression. These results are similar to those found in the literature. A recent study showed a down-regulation of KCNA5 in Ewing sarcoma that was related to the hypermethylation of the gene promoter [35]. Than and colleagues have shown that the expression of KCNA5 is inversely correlated with the progression of gastrointestinal tumours [36], which agrees with our results. Conversely, it has been reported that KCNA5 and KCNB1 gene expressions are significantly up-regulated in colon and gastric human cancers and cell lines [12]. One of the explanations for this discrepancy is possible artefacts related to the massive presence of inflammatory tumour infiltrating leucocytes (TILs) [37]. Also, the result could be also explained by tumour heterogeneity and the complexity of the tumour microenvironment [38]. In fact, it is noteworthy that the detection of KCNB1 and KCNA5 RNA expression in TILs could significantly affect Kv gene expression levels.

This preliminary interesting result prompted us to further investigate the protein expression of Kv2.1 and Kv1.5 in embedded tissues of patients with GC. Surprisingly, our processed data showed that Kv2.1 immunostaining was localised in the nucleus and the cytoplasmic compartments, whereas, Kv1.5 was present in the cytoplasmic compartment and the plasma membrane. The immunostaining of Kv2.1 and Kv1.5 was largely cytoplasmic in gastric tumour cells. Since we used highly specific antibodies with low immune-reactivity, the hypothesis of non-specific staining is quite unlikely. However, both Kv1.5 and Kv2.1 alpha subunits are able to migrate to the surface of cytoplasmic organelles or the nucleus membrane.

To the best of our knowledge, no previous studies have reported a subcellular localisation of Kv1.5 and Kv2.1 in epithelial cells. Recently, it has been demonstrated that Kv2.1 may enhance contacts between the endoplasmic reticulum and the plasma membrane in excitable cell lines [39]. More recently, an investigation on other Kv subtypes reported the localisation of Kv1.3 in the plasma membrane, the inner mitochondrial membrane, the cis-Golgi compartment and in the nuclear membrane system [24]. In addition, Kv1.1, Kv1.2 and Kv2.2 have been found to be localised in the nuclear fraction of human brain tissue and cancer cells [22, 40]. Based on the literature, Kv could also be present in the intracellular membrane network. In our study, we also highlight that Kv1.5 and Kv2.1 are ubiquitously expressed in inflammatory, peritumoral and tumoral epithelia cells. Moreover, the Kv2.1 and Kv1.5 expression profile showed a major difference between peritumoral and tumoral tissues. To our knowledge, this is the first study to have performed an immunohistochemistry analysis of Kv2.1 and Kv1.5 with the IRS scoring system. Previously, Kv1.5 has been found to be expressed in inflammatory stomach cells surrounding tumours [37]. This is the only study on Kv1.5 expression which agreed with our results. This result could explain our observation of Kv1.5 and Kv2.1 immunostaining in peritumoral gastric mucosa, with a higher intensity as compared to adjacent tumour cells. Moreover, it has been reported that Kv1.5 is expressed in diverse gastric and colon cell lines, with a higher expression in poorly-differentiated gastric cancer cells [27]. This result supports our findings showing a borderline association between the moderate expression of Kv1.5 with an earlier cancer. Nonetheless, the immunostaining of Kv2.1 was significantly associated with early GC stage. Thus, we suggest that Kv1.5 and Kv2.1 are expressed in peritumoral tissue and their altered expression manifests with a down-expression that may be involved in cancer progression. Additional functional investigations will be conducted to better expand our knowledge about the oncogenic roles of Kv2.1 and Kv1.5 channels within the nucleus, membrane and cytoplasm, and their usefulness in GC and CRC.

## Conclusion

Our results demonstrate that the expression of KCNB1 and KCNA5 is down-regulated in GC and CRC, and Kv2.1 and Kv1.5 are largely expressed in the cytoplasm and membranes of tumour gastric cells. A significant association between Kv2.1 expression and earlier stages of GC was clearly observed. This important data, although preliminary, opens up potential for Kv channels to be used as a novel tool for further exploration of GC and CRC.

## Data Availability

The datasets used in this study are available from the corresponding author.

## Abbreviations

cDNA: Complementary DeoxyriboNucleic Acid
CRC: Colorectal Cancer
DNA: DeoxyriboNucleic Acid
FFPE: Formalin-Fixed and Parffin-Embadded
GAPDH: Glyceraldehyde-3-Phosphate Dehydrogenase
GC: Gastric Cancer
IHC: Immunohistochemistry
IRS: Immuno-Reactive Score
K +: Potassium
KCNA5: Potassium Voltage-gated channel subfamily A member 5
KCNB1: Potassium Voltage-gated channel, Shab-related subfamily, member 1
Kv: Voltage-gated K+ channels
K_V_1.5: Potassium voltage-gated channel subfamily A member 5
K_V_2.1: Potassium voltage-gated channel, Shab-related subfamily, member 1
mRNA: Messenger RiboNucleic Acid
PCR: Polymerase Chain Reaction
Q-PCR: Quantitative Polymerase Chain Reaction
RNA: RiboNucleic Acid
TNM: TNM Classification of Malignant Tumors
UICC: Union for International Cancer Control
